# Community standards to facilitate development and address challenges in metabolic modeling

**DOI:** 10.15252/msb.20199235

**Published:** 2020-08-26

**Authors:** Maureen A Carey, Andreas Dräger, Moritz E Beber, Jason A Papin, James T Yurkovich

**Affiliations:** ^1^ Division of Infectious Diseases and International Health Department of Medicine University of Virginia Charlottesville VA USA; ^2^ Computational Systems Biology of Infection and Antimicrobial‐Resistant Pathogens Institute for Biomedical Informatics (IBMI) University of Tübingen Tübingen Germany; ^3^ Department of Computer Science University of Tübingen Tübingen Germany; ^4^ German Center for Infection Research (DZIF), partner site Tübingen Tübingen Germany; ^5^ Novo Nordisk Foundation Center for Biosustainability Technical University of Denmark Kemitorvet Denmark; ^6^ Department of Biomedical Engineering University of Virginia Charlottesville VA USA; ^7^ Institute for Systems Biology Seattle WA USA

**Keywords:** Computational Biology, Methods & Resources

## Abstract

Standardization of data and models facilitates effective communication, especially in computational systems biology. However, both the development and consistent use of standards and resources remain challenging. As a result, the amount, quality, and format of the information contained within systems biology models are not consistent and therefore present challenges for widespread use and communication. Here, we focused on these standards, resources, and challenges in the field of constraint‐based metabolic modeling by conducting a community‐wide survey. We used this feedback to (i) outline the major challenges that our field faces and to propose solutions and (ii) identify a set of features that defines what a “gold standard” metabolic network reconstruction looks like concerning content, annotation, and simulation capabilities. We anticipate that this community‐driven outline will help the long‐term development of community‐inspired resources as well as produce high‐quality, accessible models within our field. More broadly, we hope that these efforts can serve as blueprints for other computational modeling communities to ensure the continued development of both practical, usable standards and reproducible, knowledge‐rich models.

## Graphical abstract

Opportunities and challenges in implementing standards for modeling in computational biology are discussed using metabolic network modeling as case study.

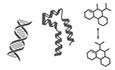

## Introduction

Systems biology uses holistic approaches to understand the networks that comprise biological systems. Computational models that attempt to represent these systems are inherently complex with many interacting components, requiring the mathematical formalization of biological phenomenon. Standardizing how these phenomena are represented is thus required to make these formalizations interpretable and accessible. Many resources—including databases, algorithms, file formats, software, and compiled “best practices”—exist to facilitate standardization (e.g., Le Novère *et al*, 2005; Waltemath *et al*, 2011; Dräger & Palsson, 2014; Ravikrishnan & Raman, 2015; Stanford *et al*, 2015; Keating *et al*, 2020), but the consistent use and application of these standards can pose a significant challenge (Ebrahim *et al*, 2015).

Here, we discuss existing standards in computational modeling in biology and when and why they are not met, building on previous efforts to assess standardization in computational systems biology (Stanford *et al*, 2015). The modeling process has two phases: model construction and simulation. Decisions about technical approaches and biological content to include in the model are made throughout both the construction and simulation processes, influencing the downstream use of the model. These implicit and explicit decisions affect the reusability; if the design decisions made during the model building process do not match well with a particular application, the quality of the simulation results will suffer. Such design decisions are influenced by a scientist's perspective, a motivating biological question, and data availability, as well as a scientist's familiarity with and access to existing resources. Manual steps of this process are particularly vulnerable to potential biases and thus are inherently irreproducible, emphasizing the role of diligent tracking of references and design decisions. Field‐defined best practices and standards can help control for or evaluate quality and facilitate iteratively cycling between construction and simulation to improve the process.

In this Commentary, we use metabolic network modeling as a case study in which to discuss the challenges to accept and implement standards. We first discuss how metabolic models are built, reviewing existing standards and their application to metabolic modeling. Next, we highlight challenges that the metabolic modeling community faces in effectively utilizing these resources, identified from a community survey. Finally, we propose an integrated set of standards which we hope will serve as a checklist to improve accessibility, interpretability, and consistency of metabolic network reconstructions. We hope that our proposed checklists will help lower the activation energy required for both experts and newcomers alike to build new reconstructions or use existing reconstructions, as well as provide a model for sustainable standardization for other systems biology fields.

## Standardization in metabolic modeling: a case study

The metabolic modeling community frequently utilizes COnstraint‐Based Reconstruction and Analysis (COBRA) methods to build and compute computational models that represent an organism's metabolic phenotype. The construction of genome‐scale metabolic network reconstructions and models is a multi‐step process that involves the reconstruction of a metabolic network, manual curation to incorporate known physiology, computation of metabolic phenotypes, and the distribution of the models and results (Box 1). The COBRA field has been led by community‐driven, open‐source software efforts (Ebrahim *et al*, 2013; Heirendt *et al*, 2019) developed to enable these kinds of analyses, building on existing systems biological standards and principles.

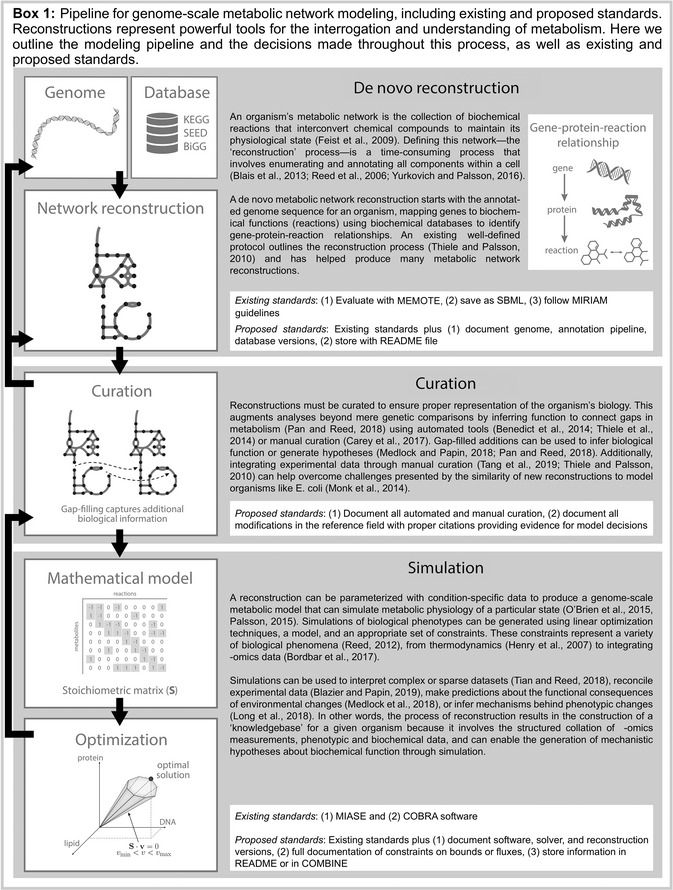

### Model structure

**Figure 1:**
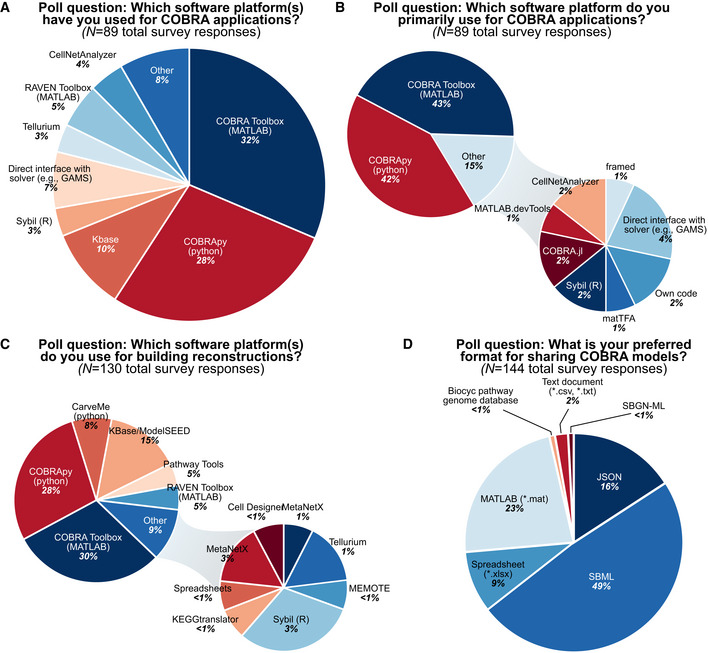
Poll results from the COBRA community survey The survey was initially compiled and released at the 5^th^ Annual Conference on Constraint‐based Reconstruction and Analysis (COBRA, October 14–16, 2018); feedback from the conference was used to refine the survey, with an updated version later shared via social media (results are shown here; raw data provided in Dataset EV1). The survey included 16 multiple‐choice and three open‐ended questions to summarize the field's use and awareness of existing standards, as well as collect community‐identified challenges. A total of 89 researchers completed the survey, representing different levels of expertise in the field; some questions permitted multiple responses (panels C and D).

SBML is the *de facto* standard file format for storing and sharing biological data and systems biology models (Keating *et al*, 2020). SBML files encode biological models in a machine‐readable format and are the most common format for editing and sharing metabolic reconstructions (Fig 1). SBML files contain lists of system components with corresponding parameters linking these components (e.g., metabolites in a reaction) and constraints (e.g., compartmentalization, reaction bounds). Saving a reconstruction as an SBML file thus inherently reinforces a set of standards. Further, the SBML field also offers several model validators and a test suite to identify nonstandard formatting in COBRA models (Table 1).

**Table 1:** Resources for using community standards and software tools

Resource	Description	Link/references
MIRIAM*	Minimum Information Required In the Annotation of biochemical Models	(Le Novère *et al*, 2005)
MIASE*	Minimum Information About a Simulation Experiment	(Waltemath *et al*, 2011)
MEMOTE	MEtabolic MOdel TEsts	https://memote.io/
COBRA‐related Google groups	Help for users of COBRApy, the python implementation of COBRA software	https://groups.google.com/forum/#!forum/cobra-pie
	Help for users of The COBRA Toolbox, the MATLAB implementation of COBRA software	https://groups.google.com/forum/#!forum/cobra-toolbox
	Discussion forum of the systems modeling community of the International Society for Computational Biology (ISCB)	https://groups.google.com/forum/#!forum/sysmod
COBRA GitHub	Repository for COBRA software, includes issue and help pages	https://github.com/opencobra/
COMBINE*	Community for coordinating standards for modeling in biology (umbrella organization for SBML, MIRIAM, MIASE, and more)	http://co.mbine.org
Kbase Help Board	Issue‐tracking system to aid users to utilize tools and datasets	https://kbase.us/help-board/
SBML Validator*	Tests the syntax and internal consistency of an SBML file	http://sbml.org/Facilities/Validator/
SBML Test Suite*	Conformance testing system to test the degree and correctness of the SBML support provided in a software package	http://sbml.org/Software/SBML_Test_Suite/
BiGG Models	Freely accessible database of GEMs	http://bigg.ucsd.edu
BioModels*	Repository of mathematical models of biological and biomedical systems	https://www.ebi.ac.uk/biomodels/
MetaNetX	Platform for accessing, analyzing and manipulating GEMs	https://www.metanetx.org
SBO terms*	Systems Biology Ontology terms are a nested classification scheme to group model components	http://www.ebi.ac.uk/sbo/

Resources developed for broad applications in computational systems biology are denoted with an asterisk; unmarked resources are specific to the COBRA field.

Ultimately, SBML is just a serialization of a particular data model and other formats for sharing models exist. The format of the serialization itself is not crucial; what matters is the format's ability to represent the necessary data structures and whether information can be unambiguously encoded and made freely accessible. These standards must be widely accepted to be easily used in multiple software tools. This pervasiveness is essential—especially for network reconstructions—where the same knowledgebase could prove useful in various applications, requiring multiple tools in a complex analysis pipeline.

### Model testing

There are different types of model evaluation processes. An important first step is to ensure a model is saved as a syntactically valid and machine‐readable SBML file with a SBML validator (Table 1); however, valid syntax does not imply biological meaning or computational correctness. Thus, a model must also be evaluated for biological sense. A recent effort to improve standardization in the COBRA community resulted in MEMOTE, a set of MEtabolic MOdel TEsts (Lieven *et al*, 2020) to increase reproducibility and model quality through model evaluation. With this tool, users can generate a report to evaluate a reconstruction, including (i) namespace of components, (ii) biochemical consistency, (iii) network topology, and (iv) versioning. MEMOTE focuses on both the technical correctness (i.e., syntax) of a model while also providing metrics that can help users to evaluate the biological correctness of the model.

Namespaces are evaluated for metabolites, genes, and reactions to check annotations for coverage, consistency, and redundancy. To check for coverage, we might ask how many metabolites have an InChI key. To ensure consistency, we evaluate if the metabolites have the correct InChI keys. Namespaces can be evaluated for redundancy by identifying how many components have additional identifiers to more thoroughly document the component. Biochemical consistency is evaluated to verify the preservation of mass and charge across both individual reactions and the entire network. MEMOTE also reports the state of the software and environment versions used by the reconstruction and during the process of testing the reconstruction. The recent development of such a community‐defined testing suite should improve the rigor of the field, particularly by tailoring general systems biology resources to our specific use cases. We encourage the community to make the use of MEMOTE an expected standard for newly published models, when applicable.

Models should also be evaluated for biological accuracy, whenever possible. Tests evaluating network topology can be used to evaluate the more subjective features of the model, by using connectedness as a proxy for inferring the scope of manual curation or the quality of a reconstruction. However, this requires such topological measures (and/or machine learning; Medlock & Papin, 2020) to be combined with biological knowledge of the system. Condition‐specific tests (often referred to as “metabolic tasks” in the COBRA field) are developed to evaluate the biological meaning of the network and attempt to represent specific biochemical experiments. Examples include production or consumption of particular metabolites given a set of constraints. Metabolic tasks can be generated for each model (including tissue‐specific models) and to evaluate iterative rounds of curation.

### Model quality and content

Many of the standards used in the COBRA field were developed by interdisciplinary teams of modelers and software developers for broad use in the computational biological modeling field (Table 1); we can use these existing resources or adapt them for use in our field as done with MEMOTE (Box 1). Minimum and recommended quality standards have been formulated and presented as a set of expectations for biological models and simulations through (i) the Minimum Information Required In the Annotation of biochemical Models (MIRIAM; Le Novère *et al*, 2005) and (ii) Minimum Information About a Simulation Experiment (MIASE; Waltemath *et al*, 2011), respectively. However, engagement in the COBRA field in particular has been modest, likely due to community members’ lack of familiarity with these resources and the challenges associated with updating these recommendations with new data types and applications. In the following sections, we discuss potential challenges facing the widespread adoption of these standards in the COBRA field and possible solutions.

## Challenges preventing the use of standards

Despite these efforts, many genome‐scale metabolic network models fail to meet minimum standards and quality metrics. Ravikrishnan and Raman found that almost 60% of models had no standardized (i.e., interpretable) metabolite identifiers, 36% could not be evaluated for mass imbalances due to unstandardized formatting, and 35% did not contain gene‐protein‐reaction associations in the SBML file (Ravikrishnan & Raman, 2015). This is a broad challenge throughout systems biology fields (Stanford *et al*, 2015). As a community, we must therefore ask why standards are not used more broadly if they enable the sharing, reuse, and evaluation of biological models and associated simulations. At the 5^th^ Annual Conference on Constraint‐based Reconstruction and Analysis (COBRA, October 14–16, 2018), we surveyed the COBRA community regarding the use of community standards. This survey identified two major causes for the lack of standardization in the COBRA field (full anonymized survey results provided in Dataset EV1).

First, the responses identified several biological phenomena that are not captured by current standards. For example, modelers of intracellular pathogen metabolism struggle to comply with nomenclature and mass balance when adding both pathogen and host biochemistry (Box 4; Carey *et al*, 2017). Similarly, it is challenging to use the correct and sufficiently detailed nomenclature for biologically relevant tautomers and polymers. While such issues will likely only be relevant in specific biological applications, it is vital that community‐adopted standards can and do evolve to address these increasingly‐common edge cases.

Second, users identified a set of novel analyses that current standards do not sufficiently support. Existing standards are inherently insufficient for novel techniques. Extensive community networks—such as modeling multiple members of the microbiota—and modeling macromolecular expression mechanisms represent current areas in metabolic modeling where some standards are currently lagging. Although standards evolve as the field progresses, they inherently cannot capture the latest cutting‐edge developments. This “lag” in standardization is not field‐specific and such cutting‐edge examples will likely only be identified in novel methods development. Both of these user‐identified limitations require community‐driven efforts to update standards as the field expands into new application areas and with novel analytic approaches.

We hypothesize that two additional factors play a role in these standardization challenges. First, biologists, modelers, and software developers are sometimes “siloed” into separate communities and with distinct motivating factors (e.g., research interests, funding mechanisms). As a result, biologists and modelers are often not aware of relevant resources generated by software developers. Our survey identified that fewer than 25% of researchers in the COBRA field were familiar with MIASE and only 56% were aware of MIRIAM; these best practices cannot be used if they are not known. In turn, biological limitations—like those discussed above—might not be relayed to software developers focusing on a standard formulation. Thus, even community‐driven efforts do not necessarily move laterally across subdisciplines. Second, as users, the lack of standardization often makes it easier to generate a novel reconstruction or analytic tool than to improve upon an existing version, further diversifying the set of existing approaches and amplifying the challenge of developing unifying standards.

## Community‐driven solutions

To remove these barriers, we suggest the field shifts to incentivize standardization by promoting model reuse and markers of quality; ultimately, this practice will improve communication among biologists, modelers, and software developers. One solution to increase the standardization in the field is to mandate compliance during the manuscript publication process—more than 85% of community‐survey responders think this should occur. However, sharing a noncompliant reconstruction or even for failing to make a reconstruction publicly available has unfortunately little consequences for authors. Incentivizing standardization through funding models is inherently challenging: Funding for science is evaluated in the short term, whereas the benefits of software or resource development are observed on a longer time scale. We can integrate funding for infrastructure (e.g., software and standards development) into applied projects (i.e., research projects). This funding approach will reflect the paired nature of these two kinds of COBRA projects.

SBML facilitates the addition of information that is specific to one particular tool or use case; once this function becomes more widely used and necessary, it can be turned into an extension package for SBML. Thus, SBML supports these edge‐case to be encoded in a standardized fashion. Hence, standards should provide such features to give enough freedom to developers of models and tools. However, this process relies on the use of such tools (e.g., SBML) and communication between the communities that design standards and the communities that use them. Such interactions could be stimulated through scientific meetings: Each conference could have a dedicated keynote presentation by a representative from the other community, followed by a panel discussion led by the presenting representative. By maintaining clear contributing instructions for the COBRA software suites, associated analysis packages, and infrastructure (e.g., SBML model format and associated API libraries), the community can update and extend standards to address edge cases.

## Recommendations for standards

In response to some of the issues and challenges outlined above, we propose a set of guidelines to help improve the accessibility, content, and quality of metabolic network reconstructions—both for those creating reconstructions/models (Box 1 and 2) and those peer‐reviewing reconstructions/models (Box 3). The suggestion of these standards was informed by panel discussions at the COBRA 2018 conference and from the community poll results (Dataset EV1), as well as previous community efforts (Stanford *et al*, 2015). Our recommendations here represent field‐specific implementation of the FAIR Data Principles (Wilkinson *et al*, 2016), a set of guidelines intended to improve reproducible research (Sansone *et al*, 2019).

**Box 2. Proposed minimum standardized content for a metabolic network reconstruction. We propose that modelers use this list as a guide to help standardize accessibility, content, and quality; however, more comprehensive documentation and more interpretable and accessible information can only improve the usability and biological relevance of the shared reconstruction. See https://github.com/maureencarey/community_standards_supplemental for tutorials demonstrating the implementation of these requirements.**

**Table:** 

**Model** Recognized naming convention o historical approach: i + *authors initials* + *number of genes in model, e.g.,* iJE660 for the *E. coli* model constructed by Jeremy Edwards with 660 genes o recommended approach: i + *species indicator *+ *iteration identifier, e.g.,* iPfal17 for *P. falciparum* published in 2017 Machine‐readable reference to organism embedded via MIRIAM annotation o full species name, including relevant identifiers if available (*e.g.,* NCBI reference genome) o taxonomy ID o strain ID, if necessary o tissue type, if necessary o URL to obtain genome Reference information o DOI o Author(s) names and contact information embedded Consistent namespace for all model identifiers
**Metabolite** Human‐readable, descriptive name (e.g., D‐Glucose) Charge (e.g., 0) Chemical formula (e.g., C6H12O6) Structural identifiers o InChI strings (if pH is known, pH‐relevant InChI ID for each metabolite) o SMILES (optional) At least one database identifier from a reliable resource, such as o MetaNetX o BiGG o KEGG Compound o ChEBI o ModelSEED o HMDb o MetaCyc
**Biochemical reaction** Human‐readable, descriptive name (e.g., phosphofructokinase) Reaction formula (e.g., ATP + L‐glutamate + ammonium ⇌ ADP + L‐glutamine + H^+^ + phosphate) At least one database identifier from a reliable resource, such as o MetaNetX o Rhea o BiGG o KEGG Reaction o ModelSEED o MetaCyc EC Number Associated genes (gene‐protein‐reaction rule, or GPR) Recommended: systems biology ontology (SBO) terms
**Biochemical reaction** Human‐readable, descriptive name (e.g., phosphofructokinase) Reaction formula (e.g., ATP + L‐glutamate + ammonium ⇌ ADP + L‐glutamine + H^+^ + phosphate) At least one database identifier from a reliable resource, such as o MetaNetX o Rhea o BiGG o KEGG Reaction o ModelSEED o MetaCyc EC Number Associated genes (gene‐protein‐reaction rule, or GPR) Recommended: systems biology ontology (SBO) terms
**Gene** Name or gene symbol DNA and/or Protein sequence ID (i.e., a mechanism for mapping this information to a sequence) o Entrez o Ensembl o UniProt o Other field‐specific database identifier Position (including chromosome, if applicable)

**Box 3: Proposed checklist for reviewers. We propose that reviewers of manuscripts that include a novel metabolic network reconstruction use this list as a guide to help standardize accessibility, content, and quality**

**Table:** 

**Reconstruction**
Availability: Is the reconstruction publicly available? o On BioModels? o Elsewhere? (optional) Is the reconstruction shared on an accessible database?
Formatting: Is the model saved in a language‐independent format (i.e., SBML)? Optional: additional formats (e.g., XLS, JSON)
Nomenclature: Does the reconstruction's name indicate a version? Does the reconstruction's name indicate an organism? Are identifiers (genes, metabolites, reactions) consistently from one namespace?
Optimization: Have Memote (preprint: Lieven *et al*, 2020) tests been run? Is the objective reaction indicated? Is evidence (i.e., references) provided for use of the objective function(s)? Are exchange, sink, and demand reactions and all necessary constraints included as defaults or in code? Can the reconstruction be instantiated without error with COBRA software?
Simulations: Are simulation parameters (objective reaction, constraints, etc.) provided in any of the following formats (include at least one): o README.md file? o COMBINE repository? o full analytic code? *e.g.,* iPython notebook or equivalent Is the COBRA software version documented? Are the solvers documented?
Manuscript: Are COBRA software efforts appropriately credited? Are previous iterations and/or other versions of the reconstruction appropriately credited? Is the model clearly referenced (i.e., with a resolvable link and identifier) in Materials & Method or in the Data Availability Statement?

First, focusing on the reconstruction process, we propose that a reconstruction metadata file is shared and includes model building information, such as the genome, database, and software versions [example README.md is provided at https://github.com/maureencarey/community_standards_supplemental, see also discussion in Box 4, or COMBINE archive in Additional file 2 of (Bergmann *et al*, 2014)]. Although this information is likely in the original manuscript, this format would link the reconstruction to the reconstruction file. The COMBINE archive also facilitates including details on gene and protein sequences, rather than mere IDs. Models should be shared on at least one publicly available repository (e.g., BioModels); because visibility increases usability, authors may want to share a model via other means as well (e.g., laboratory website, BiGG).

**Box 4: In Box 2, we identified a proposed minimum standardized content for a metabolic network reconstruction**

**Table:** 

Here, we list the associated components in the example model, iPfal19, and discuss some of the challenges in implementing these standards. This is the third iteration of the *Plasmodium falciparum* 3D7 genome‐scale metabolic network reconstruction. The original reconstruction was generated using a custom pipeline and multiple rounds of curation were conducted (Carey *et al*, 2017). iPfal19 fails to compile with several of the recommended guidelines, see notes for explanations and the Memote report for other issues. Additionally, the README file associated with this model is sparser than ideal due to the lack of documentation associated with the original curation efforts.
**Model** Recognized naming convention o iPfal19: i + *species indicator *+ *iteration identifier* model metadata (organism name, curation history, genome, authors, etc.) ORFs were called manually using proteomics and RNASeq data and compiled on the malaria parasite database; thus, no NCBI/refseq IDs (etc.) would accurately represent the genome used
**Metabolite** 100% of metabolites have a human readable, descriptive name 94% of metabolites have a charge and chemical formula^a^ 63% of metabolites have InChI strings, although because the pH for each subcellular compartment is known, these strings might not represent the appropriate species (i.e., protonation status)^b^ 100% of metabolites have an ID from BiGG (100% have BiGG‐like IDs)^c^ 91, 64.4, 68.7, 75, 47.5, and 68.4% of metabolites also have an ID from MetaNetX, KEGG Compound, ChEBI, ModelSEED, HMDb, or MetaCyc, respectively
**Biochemical reaction** 100% of reactions have a human readable, descriptive name 100% of reactions have a reaction formula 60.6% of reactions have an ID from BiGG (100% have BiGG‐like IDs)^c^ 58.6, 20.7, 0, and 23.3% of metabolic reactions have an ID from MetaNetX, KEGG Reaction, ModelSEED, and MetaCyc, respectively 27.6% of reactions have an EC Number^b^^,^ d
**Gene** Gene IDs use PlasmoDB gene nomenclature, consistent with malaria field. These IDs map to a genomic location, DNA sequence, and protein sequence on PlasmoDB.org 71.1% of reactions have GPRs

Some metabolites do not have a charge or formula associated with them including metabolites representing host or parasite proteins. If many different proteins can participate in a reaction, the reaction contains a generic reactant to represent all of the possible protein reactants. Of the 6% problematic metabolites, nearly all are proteins or aggregate metabolites.

a Not all BiGG IDs are mapped to InChI strings, EC numbers, or other useful identifiers (e.g., https://www.metanetx.org/chem_info/MNXM4217). This interferes with some Memote functionality, such as identifying duplicate reactions.

b BiGG‐like IDs are proposed new BiGG IDs consistent with the general naming approach in BiGG. For example, new IDs (pheme_fv, pheme_ap) have been created for protoheme corresponding to protoheme located in parasite‐specific compartments, the food vacuole and apicoplast, respectively, consistent with existing BiGG IDs for protoheme (http://bigg.ucsd.edu/universal/metabolites/pheme). New reactions are created when the existing BiGG reaction occurs in only one compartment but should be present elsewhere in iPfal19. For example, “PLIPA2A120pp” is a periplasmic reaction in BiGG but occurs in the cytoplasm of P. falciparum; “pp” is the suffix used to denote the periplasm so the new cytoplasmic version is named “PLIPA2A120.” Additionally, new aggregate reactions (i.e., relevant pseudoreactions) were created and named intuitively (e.g., lipid1, lipid2).

c Transporters, exchange reactions, and aggregate reactions should not have an EC number and these make up 35% of all reactions.

Second, we encourage the use of version control and specific effort to document automated and manual curation. Version control can be implemented in multiple ways, mainly through a publicly available repository that includes all iterations or by making all versions publicly available and identifiable through clear naming conventions. Further, we propose that all curation efforts be documented in the reconstruction and explicitly include a literature reference and notes in the annotations field of a reaction.

Third, we emphasize the need for MIASE requirements (Waltemath *et al*, 2011) when sharing simulation results. These data about experimental data, constraints, and versioning can be stored in a COMBINE repository or the analytic code, if publicly available. Ultimately, a standardized format (like COMBINE) could enable minor advances in COBRA software to facilitate the re‐implementation of a simulation.

## Looking ahead

Here, we have summarized existing standards in the COBRA field and identified challenges associated with both the development and compliance of software and model standards. We have proposed “checklists” for use during both the reconstruction and peer review processes that will help improve the accessibility, content, and quality of metabolic network reconstructions. Additional community‐inspired challenges and results from the COBRA community survey conducted in early 2019 are documented in Dataset EV1; we hope these examples will inspire new discussions and novel solutions.

There exist several open challenges for the field regarding the adoption of and development of new standards. We must embrace flexible standardization to facilitate their adoption and to build upon existing work. For example, although resources like MetaNetX (Moretti *et al*, 2016) and the BiGG Models database (Norsigian *et al*, 2020) facilitate the mapping of genes, reactions, and metabolites across the different namespaces, nomenclature discrepancies remain a challenge and sometimes result in redundant nonstandardized efforts. Another challenge is for community standard development to be derived from the community instead of in a top‐down manner. While this organizational structure is currently in effect for the SBML community, it only functions if there is community participation—we need those who use the standards (i.e., modelers) to raise their hands and participate in the decision making process.

Ultimately, community adherence to standards will improve modeling reproducibility and better document the reconstruction process. We hope that the community embraces existing standards and our community‐driven suggestions moving forward—both during the preparation of manuscripts and during the peer review process—and anticipate that compliance will increase the rigor of the field while simultaneously making it easier for scientists from other disciplines to build and use metabolic models.

## Conflict of interest

The authors declare that they have no conflict of interest.

## Supporting information



Dataset EV1
